# Outbreak of Highly Pathogenic Avian Influenza A(H5N1) Virus in Seals, St. Lawrence Estuary, Quebec, Canada^1^

**DOI:** 10.3201/eid3006.231033

**Published:** 2024-06

**Authors:** Stéphane Lair, Louise Quesnel, Anthony V. Signore, Pauline Delnatte, Carissa Embury-Hyatt, Marie-Soleil Nadeau, Oliver Lung, Shannon T. Ferrell, Robert Michaud, Yohannes Berhane

**Affiliations:** Université de Montréal, St. Hyacinthe, Quebec, Canada (S. Lair, L. Quesnel, P. Delnatte, S.T. Ferrell);; Canadian Food Inspection Agency, Winnipeg, Manitoba, Canada (A.V. Signore, C. Embury-Hyatt, O. Lung, Y. Berhane);; Ministère de l’Agriculture, des Pêcheries et de l’Alimentation du Quebec, St. Hyacinthe (M.-S. Nadeau);; Groupe de recherche et d’éducation sur les mammifères marins, Tadoussac, Quebec, Canada (R. Michaud).

**Keywords:** influenza, avian influenza, respiratory infections, viruses, encephalitis, H5N1, highly pathogenic avian influenza, HPAI, pinnipeds, seals, St. Lawrence Estuary, unusual mortality event, Quebec, Canada

## Abstract

We describe an unusual mortality event caused by a highly pathogenic avian influenza (HPAI) A(H5N1) virus clade 2.3.4.4b involving harbor (*Phoca vitulina*) and gray (*Halichoerus grypus*) seals in the St. Lawrence Estuary, Quebec, Canada, in 2022. Fifteen (56%) of the seals submitted for necropsy were considered to be fatally infected by HPAI H5N1 containing fully Eurasian or Eurasian/North American genome constellations. Concurrently, presence of large numbers of bird carcasses infected with HPAI H5N1 at seal haul-out sites most likely contributed to the spillover of infection to the seals. Histologic changes included meningoencephalitis (100%), fibrinosuppurative alveolitis, and multiorgan acute necrotizing inflammation. This report of fatal HPAI H5N1 infection in pinnipeds in Canada raises concerns about the expanding host of this virus, the potential for the establishment of a marine mammal reservoir, and the public health risks associated with spillover to mammals.

Nous décrivons un événement de mortalité inhabituelle causé par un virus de l’influenza aviaire hautement pathogène A(H5N1) clade 2.3.4.4b chez des phoques communs (*Phoca vitulina*) et gris (*Halichoerus grypus*) dans l’estuaire du Saint-Laurent au Québec, Canada, en 2022. Quinze (56%) des phoques soumis pour nécropsie ont été considérés comme étant fatalement infectés par le virus H5N1 de lignées eurasiennes ou de réassortiment eurasiennes/nord-américaines. Un grand nombre simultané de carcasses d’oiseaux infectés par le H5N1 sur les sites d’échouement a probablement contribué à la contamination de ces phoques. Les changements histologiques associés à cette infection incluaient : méningo-encéphalite (100%), alvéolite fibrinosuppurée et inflammation nécrosante aiguë multi-organique. Cette documentation soulève des préoccupations quant à l’émergence de virus mortels, à la possibilité d’établissement de réservoirs chez les mammifères marins, et aux risques pour la santé publique associés aux propagations du virus chez les mammifères.

Sporadic outbreaks of influenza A virus (IAV) infections have been reported in pinnipeds in the United States and Europe, most commonly in harbor (*Phoca vitulina*) and gray (*Halichoerus grypus*) seals (*1*–*3*). Characterization of the IAV-associated subtypes has suggested an avian-variant origin, thought to occur through cross-species transmission or spillover from wild aquatic birds (*4*,*5*). The reported IAV outbreaks in pinnipeds have mainly caused fatal respiratory diseases (*6*,*7*). Harbor seals seem to be particularly susceptible to IAV infections, and factors such as close contact with wild birds and mammalian adaptations of the virus subtypes have been suggested as drivers in establishing a potential reservoir of IAV in marine mammals (*5*,*8*).

Although epidemics of IAV have been reported since the late 1970s in seals on the North American Atlantic coast, fatal influenza virus infections have not been documented in marine mammals from the St. Lawrence Estuary and Gulf, Quebec, Canada. Moreover, despite the documented high seroprevalence of influenza virus A and B in Canada seal populations (*9*), seal deaths caused by an influenza infection were initially reported in Canada when a novel low-pathogenicity avian IAV (H10N7) caused fatal bronchointerstitial pneumonia in a harbor seal in British Columbia (*10*). 

A wild gull found dead in eastern Canada in November 2021 was confirmed to be infected by HPAI H5N1 clade 2.3.4.4b A/goose/Guangdong/1/1996 (Gs/GD) lineage (*11*). That novel virus subtype was likely introduced by wild birds that carried it across the Atlantic Ocean through pelagic routes or during direct winter migration (*11*). The virus spread rapidly across North America and reassorted with North American lineage IAVs, causing unprecedented outbreaks in many species of wild birds and commercial and backyard poultry flocks and spillover to several species of wild terrestrial mammals (*12*–*16*).

During summer 2022, deaths of harbor and gray seals caused by H5N1 clade 2.3.4.4b virus infection were confirmed in eastern Quebec and on the coast of Maine, USA, prompting the US National Oceanic and Atmospheric Administration to declare an unusual mortality event for Maine harbor and gray seals (*17*). HPAI infections have also recently been reported as the cause of death of harbor seals in the North Sea (H5N8 virus) (*18*), gray seals in the Baltic Sea (H5N8 virus) (*19*), South American sea lions (*Otaria flavescens*) in Peru (*20*) and Chile (*21*), and other marine mammals in Peru (*22*). Given the emergence of this virus in marine mammals in the St. Lawrence Estuary and public health concerns associated with mammalian spillover of avian IAV, we aimed to describe the 2022 outbreak of HPAI H5N1 affecting pinniped species, emphasizing epidemiologic data and pathology findings.

## Material and Methods

### Stranding Data Analysis

We obtained stranding data from the archives of the Quebec Marine Mammal Emergency Response Network, which monitors mortality and morbidity of marine mammals in the St. Lawrence River, Estuary and Gulf (48°23′N, 69°07′W). We compared the number of stranded (dead, ill, or injured) harbor seals, gray seals, and seals of unspecified species during the second and third quarters of 2022 (April 1–September 30) with the average of the 10 previous years for the same period using a 1-sample *t*-test. We evaluated goodness-of-fit of the stranding distribution of 2012 to 2021 with a Shapiro-Wilk test for both groups of seals. We considered distribution normal if p>0.05. We combined seals of unidentified species with harbor seals for this comparison to control for the differences in species identification rates in 2022 compared with previous years (data not shown).

### Postmortem Examination

We based our selection of carcasses to be examined on the state of decomposition (*23*) and field access. All carcasses were submitted frozen and, after thawing, were examined by veterinary pathologists with experience in marine mammal pathology. Animals were classified into 2 age groups, <1 year old or adult, on the basis of total length (*24*). Animals with no evidence of muscular or fat depletion were considered to be in good nutritional condition. Tissue samples of major organs (lung, heart, kidney, brain, intestines, lymph nodes, liver, spleen, pancreas, tongue, adrenal gland, esophagus, bladder, stomach, thyroid gland, mammary gland, and thymus) were processed for histopathologic evaluation by light microscopy using standard laboratory procedures. For each necropsy case, separate nasal and rectal swab samples were collected using a sterile polyester-tipped plastic applicator (UltiDent Scientific, https://www.ultident.com) placed in UT medium (Micronostyx, https://micronostyx.com). In addition, rectal and nasal swab samples were collected from seals found stranded for which the carcass could not be examined either because of poor preservation state or logistical limitations. All samples were first tested for IAV by PCR at the provincial Animal Health Laboratory (Laboratoire de santé animale, Ministère de l’Agriculture, des Pêcheries et de l’Alimentation du Québec; MAPAQ). All IAV H5–positive samples were subsequently sent to the National Centre for Foreign Animal Disease (NCFAD) laboratory (Winnipeg, MB, Canada) for confirmatory testing. Samples of brain or lung were also submitted for PCR for cases that had lesions suggestive of IAV but tested negative on swab samples.

### RNA Extraction, Reverse Transcription PCR, and Virus Isolation

Both laboratories (MAPAQ and NCFAD) used the same methods for RNA extraction and PCR. Total RNA was extracted from clinical specimens (swabs and tissues) and virus isolates using the MagMax AM1836 96 Viral RNA Isolation Kit (ThermoFisher Scientific, https://www.thermofisher.com) according to manufacturer recommendations, using the KingFisher Duo Prime, KingFisher Flex, or Apex platforms (ThermoFisher Scientific). Spiked enteroviral armored RNA (Asuragen, https://asuragen.com) was used as an exogenous extraction and reaction control. The extracted RNA samples were tested for IAV genomic material by using matrix gene–specific real-time reverse transcription PCR. Samples positive with the matrix primer set underwent repeat PCR with H5- and H7-specific primer sets, as described previously (*25*,*26*). Cycle threshold values <36.00 were considered positive and values 36.00–40.00 suspicious. For virus isolation, PCR-positive samples were inoculated into 9-day-old embryonated specific pathogen-free chicken eggs via the allantoic route.

### Nanopore Sequencing and Genome Assembly

To determine the clade, lineage, and clusters of each positive sample, the full genome segments of IAVs were amplified directly from clinical specimens or isolates using reverse transcription PCR, as described previously (*27*). Nanopore sequencing was performed on a GridION sequencer (Oxford Nanopore, https://nanoporetech.com) with an R9.4.1 flowcell after library construction using the rapid barcoding kit (SQKRBK004 or SQK-RBK110.96). The raw nanopore signal data was basecalled and demultiplexed with Guppy version 5.1.12 (Oxford Nanopore) using the high accuracy or super-accurate basecalling model on each run. Basecalled nanopore reads were analyzed and assembled with a BLAST search (https://blast.ncbi.nlm.nih.gov) of Iterative Refinement Meta-Assembler assembled genome segment sequences against all sequences from the National Center for Biotechnology Information Influenza Virus Sequence Database (n = 959,847) (https://www.ncbi.nlm.nih.gov/genomes/FLU/Database/nph-select.cgi) and influenza virus sequences from the 2021–2022 HPAI H5N1 outbreaks. We deposited whole-genome sequences of the HPAI H5N1 viruses from seals into the GISAID database (https://www.gisaid.org; accession nos. EPI_ISL_18916928–37).

### Phylogenetic Analyses

We combined the HPAI H5N1 genomes sequenced from seals (10 samples) with 40 closely related sequences (as determined by BLAST similarity search) collected from wild birds during April–September 2022. We trimmed individual viral segments (polymerase basic 1 and 2, polymerase acidic, hemagglutinin, nucleoprotein, neuraminidase, matrix, nonstructural) of regions flanking the open reading frames and concatenated. We removed duplicate sequences from the dataset, leaving 43 complete viral genomes, totaling 13,112 nt in length, which we aligned using MAFFT version 7.49 (https://mafft.cbrc.jp). We used that alignment to estimate a time-scaled phylogenetic tree using BEAST version 1.10.4 (*28*). We performed tree estimations under the best fitting model of nucleotide substitution as determined by ModelFinder (generalized time-reversible model, empirical base frequencies, invariant sites, 4-category gamma distribution of rate heterogeneity) (*29*), a relaxed molecular clock with log-normal distribution, and a Gaussian Markov Random Field Bayesian skyride tree prior (*30*). We also ran 2 independent Markov chain Monte Carlo chains (200 million steps, sampled every 20,000 steps), discarding the first 10% of samples from each chain as burn-in, and assessed the chains for convergence (based on effective sample size >200) using Tracer version 1.7.2 (*31*). We combined post–burn-in samples using LogCombiner version 1.10.4 and produced maximum clade credibility (MCC) trees by using TreeAnnotator version 1.10.4 (*28*).

### Immunohistochemistry

For immunohistochemistry (IHC), we quenched paraffin tissue sections for 10 minutes in aqueous 3% hydrogen peroxide. We then retrieved epitopes using proteinase K for 15 minutes and rinsed. The primary antibody applied to the sections was a mouse monoclonal antibody specific for IAV nucleoprotein (F26NP9, produced in-house) used at a 1:5,000 dilution for 30 minutes. We visualized the primary antibody binding by using a horseradish peroxidase labeled polymer, the Dako EnVision+ system (anti-mouse) (Agilent, https://www.agilent.com), reacted with chromogen diaminobenzidine. We then counterstained the section with Gill hematoxylin.

## Results

### Stranding Data

**Figure 1:**
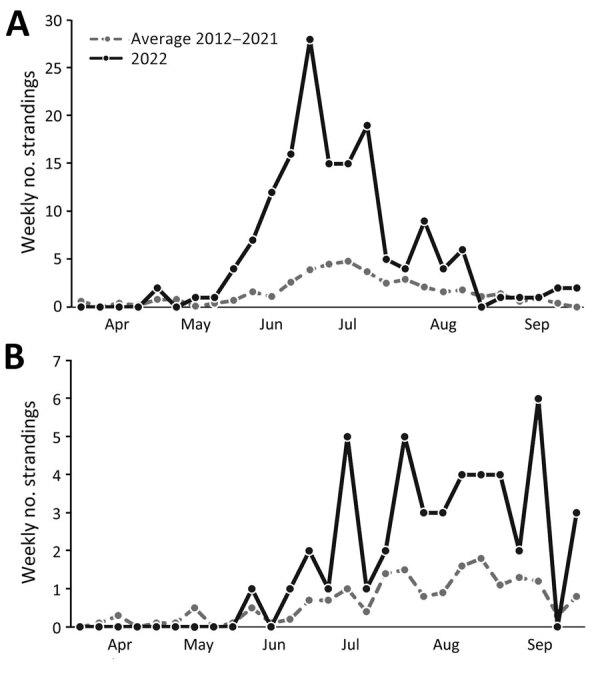
Weekly occurrences of stranded seals in an outbreak of highly pathogenic avian influenza A(H5N1) virus in seals, the St. Lawrence Estuary and Gulf, Quebec, Canada. Graphs compare strandings during April 1–September 30, 2022, with the average number of strandings over the previous 10 years (2012–2021) during the same quarters. A) Harbor seals (*Phoca vitulina*) and seals of undetermined species; B) gray seals (*Halichoerus grypus*).

During April 1–September 30, 2022, a total of 209 dead or sick seals were reported in the waters bordering Quebec: 127 harbor seals, 47 gray seals, 6 harp seals (*Pagophilus groenlandicus*), 1 hooded seal (*Cystophora cristata*), and 28 seals of undetermined species. The number of dead or sick stranded harbor seals and seals of unknown species combined during that period of time (n = 55) was 3.7 times higher than the average annual number in the previous 10 years for the same period (n = 41.6), representing a statistically significant increase in number of strandings (*t* = 5.55, d.f. = 9; p<0.001) (Figure 1, panel A). A statistically significant increase of similar magnitude was noted for the number of gray seals found dead or sick during the second and third quarters of 2022 (47 in 2022 compared with an average of 12 in 2012–2021; *t* = 4.35, d.f. = 9; p = 0.002) (Figure 1, panel B). We observed no increase in mortality or morbidity for the other species of pinnipeds compared with the previous 10 years.

### Descriptive Epidemiology and Necropsy Findings

Carcasses of 22 harbor seals, 3 gray seals, 1 harp seal, and 1 hooded seal found during April 22–October 6, 2022, were submitted for postmortem examination. On the basis of molecular detection of H5 and presence of lesions suggestive of IAV infection, HPAI H5N1 was identified as cause of death of 14 harbor seals and 1 gray seal (56%) (Table 1). Combined nasal and rectal swab samples were obtained on the shore from an additional 11 harbor seal and 1 gray seal carcasses, and HPAI H5N1 was identified in 6 of the harbor seals (50% of those sampled) (Table 1).

**Table 1:** Percentages of stranded seals tested that were infected by HPAI A(H5N1) in the St. Lawrence Estuary, Quebec, Canada, April 22–October 6, 2022*

Species and sampling	% Infected (no. infected†/no. tested)
Harbor seal (*Phoca vitulina*)	
Full postmortem examination	64 (14/22)
Nasal/rectal field swab	55 (6/11)
Total	61 (20/33)
Grey seal (*Halichoerus grypus*)	
Full postmortem examination	33 (1/3)
Nasal/rectal field swab	0 (0/1)
Total	25 (1/4)
Hooded seal (*Cystophora cristata*)	
Full postmortem examination	0 (0/1)
Harp seal (*Pagophilus groenlandicus*)	
Full postmortem examination	0 (0/1)
Total pinnipeds	
Full postmortem examination	56 (15/27)
Nasal/rectal field swab	50 (6/12)
Total	54 (21/39)

*Postmortem and field swab samples were from different individual strandings. HPAI, highly pathogenic avian influenza. †Infections were determined on the basis of epidemiologic data, presence of suggestive lesions, and PCR testing using H5-specific primers.

**Figure 2:**
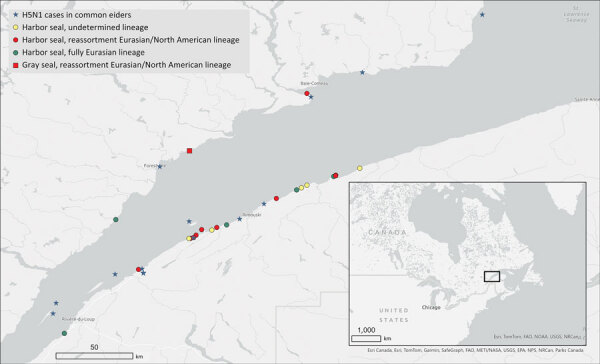
Geographic locations of stranded, dead, or sick seals infected by highly pathogenic avian influenza A(H5N1) virus during the 2022 outbreak in the St. Lawrence Estuary, Quebec, Canada. The locations of harbor seals (*Phoca vitulina*) gray seals (*Halichoerus grypus*) and detected H5N1 lineages are marked as are the documented outbreaks in common eider (*Somateria mollissima*) colonies. Inset shows study location in a map of eastern Canada and US Midwest and Northeast.

All 21 infected seals were found during May 30–July 8, 2022, in the estuarine segment of the St. Lawrence waterway, mainly on the south shore, between the towns of Baie-Comeau (49.2213°N, 68.1504°W) and Notre-Dame-du-Portage (47.7630°N, 69.6096°W) (Figure 2). Infections were detected in both <1 year old and adult seals with no age predilection. All the infected adult harbor seals (n = 9) were female, and 6 had evidence of recent parturition (active lactation, asymmetric uterine horns without the presence of a fetus, or both). The infected adult gray seal was male. There were 3 male and 7 female (and 1 nondetermined) infected <1 year old seals (Table 2). One of the infected seals was found alive with profound lethargy and neurologic signs. In addition, anecdotal observations of weak and dyspneic harbor seals were reported during the outbreak.

**Table 2:** Demographic data and results of immunohistochemistry and molecular testing performed in seals infected by HPAI A(H5N1) during the 2022 outbreak in the St. Lawrence Estuary, Quebec, Canada*

Case no.	Date found	Species†	Age group/sex	AIV IHC	Initial PCR at MAPAQ			Confirmatory PCR at NCFAD		Sample type‡	Lineage	IAV histologic lesions
					Matrix	H5		Matrix	H5			
217719	May 30	Harbor seal	Adult/F	+	+	+		+	+	Swab	EU/NA	Yes
216969	June 7	Harbor seal	<1 y	+	+	+		+	+	Swab	EU	Yes
216970	June 7	Harbor seal	Adult/F	+	+	+		+	+	Swab	EU	Yes
216971	June 8	Harbor seal	Adult/F	-	+/−	+		+	+	Swab	EU	Yes
216947	June 10	Harbor seal	Adult/F	+	+	+		+	+	Brain	EU/NA	Yes
216983	June 11	Harbor seal	Adult/F	NP	+	+		+	+	Swab	EU/NA	NP§
216985	June 13	Harbor seal	<1 y/F	NP	+	+		+	+	Swab	NP	NP§
216972	June 14	Harbor seal	<1 y/F	+	+	+		+	+	Swab	EU	Yes
217671	June 14	Harbor seal	Adult/F	+	+	+		+	+	Swab	EU	Yes
216987	June 19	Harbor seal	Adult/F	NP	+	+		+	+	Swab	EU/NA	NP§
216988	June 20	Harbor seal	<1 y/F	+	+	+		+	+	Swab	EU/NA	Yes
216989	June 20	Harbor seal	Adult/F	NP	+	+		+	+	Swab	NP	NP§
217670	June 20	Harbor seal	<1 y/F	+	+	+		+	+	Swab	EU/NA	Yes
216973	June 22	Harbor seal	Adult/F	NP	+	+/−		+	+	Brain	NP	Yes
216974	June 22	Harbor seal	<1 y/F	+	+/−	+		+	+	Brain	EU/NA	Yes
217794	June 24	Gray seal	Adult/F	+	+	+		+	+	Swab	EU/NA	Yes
217642	June 26	Harbor seal	<1 y/F	+	+	+		+	+	Swab	EU/NA	Yes
217665	June 26	Harbor seal	<1 y/F	+	+/−	+		+	+	Swab	EU/NA	Yes
217667	June 26	Harbor seal	<1 y/F	NP	+	+		+	-	Lung	NP	Yes
217611	July 7	Harbor seal	<1 y/F	NP	+	+		+	+	Swab	EU/NA	NP§
217612	July 8	Harbor seal	<1 y/ND	NP	+/−	+/−		+	+	Swab	NP	NP§

*EU, fully Eurasian lineage; EU/NA, reassortment between Eurasian and North American lineages; HPAI, highly pathogenic avian influenza; IAV, influenza virus; IHC, immunohistochemistry; MAPAQ, Ministère de l’Agriculture, des Pêcheries et de l’Alimentation du Québec; NCFAD, National Center for Foreign Animal Disease; ND, not determined; NP, not performed. –, negative; +, positive; +/−, suspicious. †Harbor seal, *Phoca vitulina*; gray seal, *Halichoerus grypus*. ‡Swab indicates combined nasal and rectal swab samples. Frozen lung or brain were submitted when swab testing results were negative. §Cases for which only swabs collected in the fields were available; no necropsy performed.

**Figure 3:**
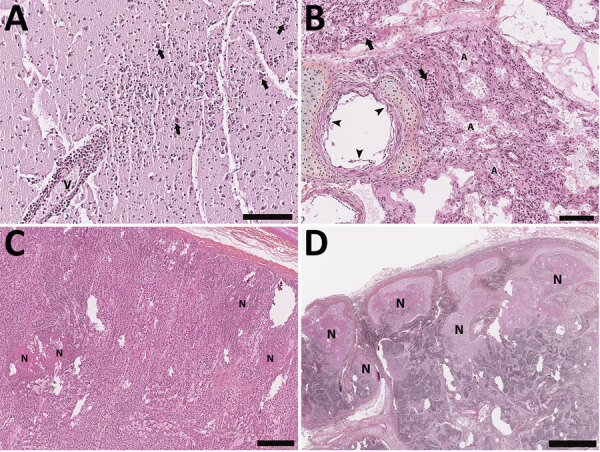
Histology section of thawed, formalin-fixed tissues from harbor seals (*Phoca vitulina*) infected by highly pathogenic avian influenza A(H5N1) virus in the St. Lawrence Estuary, Quebec, Canada, 2022. Hematoxylin phloxine saffron stain. A) Brain tissue from a young (<1 year old) female seal. The Virchow-Robin space around a vessel (V) is infiltrated by numerous layers of polymorphonuclear cells. Several neurons have a condensed hyperacidophilic cytoplasm indicative of necrosis and are often associated with satellitosis (arrows). A focally extensive infiltration of the neuropil by neutrophils and glial cells is also present. Scale bar indicates 100 µm. B) Lung from a young (<1 year old) female seal. The alveolar (A) and vascular (arrow) lumens contain numerous, often degenerate, polymorphonuclear cells. The alveolar walls are infiltrated by numerous inflammatory cells composed of neutrophils and mononuclear cells. The epithelial cells bordering the small bronchi are often necrotic. Scale bar indicates 100 µm. C) Adrenal gland of an adult female seal. Multifocal foci of necrosis are present in the cortical zone (N). Scale bar indicates 300 µm. D) Lymph node from an adult female seal. Marked multifocal to coalescing necrosis of the lymphoid tissues in the cortical region are noted (N). Scale bar indicates 1 mm.

Carcasses were attributed decomposition scores (*21*), varying from code 2–2.5 (fresh to mild decomposition) in 12 cases to code 3 (moderate decomposition) for 3 cases. Twelve seals were in excellent nutritional condition, 2 were thin, and 1 was emaciated. Postmortem findings (Table 3) showed notable gross lesions limited to lymphadenomegaly (submandibular and mesenteric lymph nodes), red-tinged foam in the tracheal lumen, and pulmonary congestion. Relevant histologic lesions (Table 3) included acute multifocal to diffuse mixed meningoencephalitis (Figure 3, panel A), characterized by a predominantly neutrophilic infiltrate with lymphocytes in Virchow-Robin spaces, the meninges, the submeningeal neuropil. Neuronal necrosis, satellitosis, and gliosis were also regularly observed. Acute fibrinosuppurative alveolitis, consisting of neutrophils with fibrinous aggregates in the alveolar lumen (Figure 3, panel B), was observed in 9/15 seals. Interstitial pneumonia, characterized by a mild to moderate mixed inflammatory infiltrate within the alveolar septa, was seen either concurrently or distinctively from the alveolitis. In addition to the inflammatory changes, alveolar emphysema, mild acute alveolar damage with hyaline membranes, and necrotic type 1 pneumocytes were sometimes present. Six animals presented multifocal necrotic foci of the adrenal cortex with infiltrates of degenerate neutrophils (Figure 3, panel C). Acute necrotizing thymitis, lymphadenitis, and splenitis were also observed and consisted of multifocal to coalescing zones of necrosis, mainly centered on cortical lymphoid follicles along with extensive lymphoid depletion (Figure 3, panel D). Lymph nodes were often reactive with an accumulation of histiocytic cells in the lymphatic trabeculae and medullary sinus, macrophages in the germinal centers, and an infiltration of the subcapsular sinus with granulocytes and macrophages. Other microscopic changes were seen in 6 cases with mild membranous glomerulonephritis, 4 cases with necrotizing fibrinous hepatitis of variable severity, 1 case of mild multifocal myocarditis with degenerate myofibers, and 1 animal with mild neutrophilic perivascular infiltration in the perimysium.

**Table 3:** Microscopic lesions documented in harbor (*Phoca vitulina*) and gray (*Halichoerus grypus*) seals infected by HPAI A(H5N1) during the 2022 outbreak in the St. Lawrence Estuary, Quebec, Canada

Microscopic lesions	% Seals affected (no. with lesions/no. examined)
Brain	
Meningoencephalitis	100 (15/15)
Predominantly neutrophilic	80 (12/15)
Predominantly lymphoplasmacytic	20 (3/15)
Lung	
Pulmonary inflammatory changes	73 (11/15)
Acute multifocal fibrinosuppurative alveolitis	60 (9/15)
Acute interstitial pneumonia	53 (8/15)
Acute alveolar damage	40 (6/15)
Alveolar emphysema	27 (4/15)
Adrenal gland	
Adrenocortical necrosis	60 (6/10)
Thymus	
Acute multifocal necrotizing thymitis	50 (2/4)
Lymph nodes	
Reactional lymph nodes	43 (6/14)
Acute multifocal necrotizing lymphadenitis	36 (5/14)
Kidney	
Multifocal membranous glomerulonephritis	40 (6/15)
Liver	
Necrotizing hepatitis	29 (4/14)
Spleen	
Necrotizing splenitis	21 (3/14)
Muscle	
Neutrophilic infiltration of the perimysium	9 (1/11)
Heart	
Multifocal myocarditis	7 (1/14)

**Figure 4:**
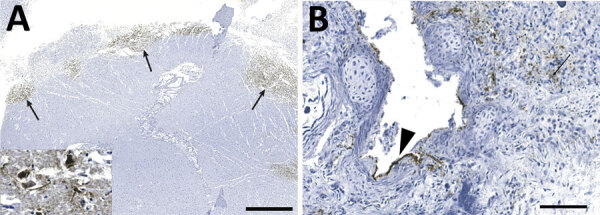
Detection of influenza virus antigen by immunohistochemistry in brain (A) and lung (B) of harbor seals (*Phoca vitulina*) infected by highly pathogenic avian influenza A(H5N1) virus, St. Lawrence Estuary, Quebec, Canada, 2022. A) Brain tissue. Multifocal areas of intense immunostaining (arrows) with staining of all structures are seen in the affected area, including neurons and neuropil (inset). Scale bar indicates 2 mm. B) Lung tissue. Positive immunostaining can be observed within alveolar septae (arrow) and in bronchiolar epithelial cells (arrowhead). Scale bar indicates 80 µm.

Immunoreactivity for IAV antigen was observed in tissues from 12 of the 13 cases that were tested (Table 2). Variable abundance of antigens, from mild to extensive, was detected in different tissues, including the neuropils and neurons (Figure 4, panel A), pulmonary alveolar septa and glandular bronchial cells (Figure 4, panel B), renal glomeruli, spleen, pancreas, liver, vascular walls of skeletal muscle, lymph nodes, tracheal vessels, and adrenal glands. Those antigens were often associated with, but not limited to, necrotic foci.

### Virology Assessment

**Figure 5:**
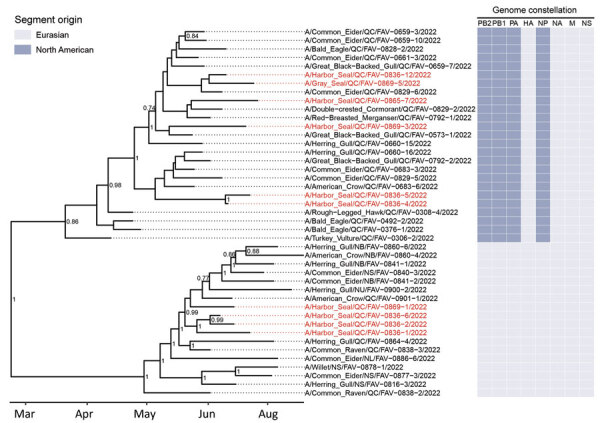
Time-scaled phylogenetic tree of 43 highly pathogenic avian influenza A(H5N1) virus clade 2.3.4.4b complete viral genomes from seals and wild birds in the St. Lawrence Estuary, Quebec, Canada, 2022. Tree was inferred by Bayesian analysis. Red text indicates seal-derived sequences and black text indicates wild bird–derived sequences. Posterior probability values >0.70 are displayed at the tree nodes. The genome constellation for each sequence (i.e., the compliment of Eurasian and North American derived genome segments) is presented to the right of the tree tips. HA, hemagglutinin; M, matrix; NA, neuraminidase; NP, nucleoprotein; NS, nonstructural; PA, polymerase acidic; PB, polymerase basic.

The presence of IAV H5 RNA was confirmed by PCR in 15 necropsied seals and in 6 seals that were swabbed in the field (n = 21) (Table 2). All samples were negative for H7. All 15 necropsied seals had lesions suggestive of IAV on histology. In 4 cases, NCFAD confirmatory PCRs for H5 performed on combined rectal/nasal swab samples were negative but H5 RNA was detected by subsequent PCR on frozen lung or brain tissues. Virus isolation (using embryonated specific pathogen-free chicken eggs) was successful in 16 of those 21 cases. The 16 isolates were sequenced to determine the subtype and lineage of the H5 virus. All isolates belonged to Gs/GD lineage H5N1 clade 2.3.4.4b. Five isolates had fully Eurasian gene segments similar to the Newfoundland-like clade 2.3.4.4b H5N1 viruses that emerged in Canada in late 2021. The other 11 isolates were reassortant H5N1 viruses containing gene segments polymerase basic 1 and 2, polymerase acidic, and nucleoprotein, belonging to the North American lineage IAVs and hemagglutinin, neuraminidase, matrix, and nonstructural gene segments belonging to the Newfoundland-like clade 2.3.4.4b H5N1 viruses. Accordingly, the phylogenetic tree estimated from those sequences is bifurcated at the root between fully Eurasian and reassortant viruses (Figure 5). In both the fully Eurasian and reassortant lineages, sequences derived from wild birds are ancestral to the (well-supported and monophyletic) clades that include seals. That finding provides strong support for independent bird-to-seal spillover events for each viral lineage, rather than a single spillover and subsequent reassortment event in seals.

## Discussion

Investigation of increased deaths among the pinniped populations of the St. Lawrence Estuary and Gulf during the summer of 2022 detected HPAI H5N1 infections in harbor and gray seals. All necropsied seals that were positive for H5 by PCR also manifested histologic lesions consistent with IAV infection. The demonstration by IHC that IAV antigens were often associated with necrotic and inflammatory lesions further supports a causal relationship between this virus, the observed lesions, and the animal deaths. The almost 4-fold increase in summer deaths among harbor and gray seals compared with historic data could reasonably be explained, at least in part, by an HPAI H5N1 outbreak affecting those populations of seals.

Even though we observed good agreement between the different diagnostic modalities, a few discrepancies between the results are worth noting (Table 2): IHC failed to detect 1 infected seal; 4 cases that were considered only suspected based on an initial matrix or H5 PCRs by MAPAQ were later confirmed positive by NCFAD; and PCR results for combined nasal/rectal swab samples had a false-negative rate of 27% (4/15). Such differences in diagnostic results highlight the importance of a multistep diagnostic methodology to confirm avian IAV cases and demonstrate that surveillance efforts solely based on nasal/rectal swab sample PCR results likely grossly underestimate the actual prevalence of infection.

Most of the examined animals were in good nutritional condition with only minor macroscopic changes, except for noticeable lymphadenomegaly. However, some macroscopic changes possibly were overlooked because of postmortem and freezing artifacts. Inflammatory and necrotic changes were detected in several organs, including brain, lungs, adrenal glands, liver, and lymph. The central nervous system (100%) and the lungs (73%) were the most commonly affected organs. That neurotropism and respirotropism are similar to what has been reported in birds (*32*) and in mesocarnivores infected with HPAI H5N1 (*12*,*15*), as well as in 3 harbor seals from the North Sea near Germany infected with HPAI H5N8 (*18*). The predominantly neutrophilic nature of the meningoencephalitis is unusual for a viral infection and indicative of a very acute infection.

The route of transmission and the viral pathogenesis for HPAI infection have not been established in pinnipeds. In most mammals, including humans, IAVs are transmitted through inhaling aerosols or respiratory fomites from an infected individual and replicate primarily in respiratory epithelium. Previous reports of infections with low-pathogenicity avian influenza in pinnipeds are consistent with the same pathophysiology as with stranded sick seals displaying respiratory signs, whereas dead seals showed necrotizing hemorrhagic bronchitis and alveolitis (*6*,*7*). However, the clinical manifestations and postmortem lesions seen in various mammal species affected by HPAI H5N1, including the seals from this study, indicate a clear neurotropism (*12*,*15*,*16*). Neurologic signs, encephalitis, and a high load of viral antigens and RNA in the brain all point toward a different pathogenesis and possibly a different route of inoculation. 

Most mammal species that have been reported to be infected by HPAI H5N1 are carnivores that are likely to prey or scavenge on wild birds. Consequently, ingestion of birds infected with HPAI is presumed to be the most likely source of infection in free-ranging wild mesocarnivores (*12*,*15*) and in sporadic cases of infected domestic or captive wild carnivores (*33*,*34*). That route of transmission, which has been proven successful by red foxes being experimentally infected with HPAI H5N1 clade 2.2 after eating infected bird carcasses (*35*), is also plausible in gray seals because this species is known to prey on sea birds, making a weakened bird or infected carcass a potential source of infection. However, that pathway of infection is unlikely for harbor seals because they are not known to predate or scavenge on birds. Consequently, transmission through environmental exposure, such as accidentally ingesting feces or feathers from infected birds, drinking fecal-contaminated water, or inhaling aerosols or respiratory fomites from infected avian carcasses, should be considered for harbor seals. All harbor seals that died of HPAI H5N1 in this population were found in relatively close proximity to islands used as pupping grounds. Some of those islands also harbor breeding colonies of marine birds, such as the common eiders (*Somateria mollissima*). Numerous cases of death caused by HPAI H5N1 were documented in those marine bird colonies during the same time period (Canadian Wildlife Health Cooperative database, unpub. data). Anecdotal observations of seals hauling out on sites where carcasses of eiders were present were reported during the outbreak. Those observations would support the hypothesis that harbor seals were exposed to HPAI H5N1 by close contact with infected marine birds. The infected harbor seals were also either newborn or female and in breeding age, supporting the link between the infections and contact with marine birds at pupping sites. 

The fact that infections by HPAI H5N1 was documented in only 1 gray seal, even if that species was abundant in the St. Lawrence Estuary during the period of the outbreak (*36*), suggests either that gray seals are less sensitive to this virus or that they were less exposed to the virus compared with harbor seals. Indeed, in contrast to harbor seals that give birth to their pups in the spring/summer, the gray seal pupping season is in the winter. Therefore, that species is less likely to be in contact with potentially infected nestling colonial birds. The absence of documentation of cases of infections in seals in the St. Lawrence Gulf, where both species of seals are abundant (*36*), might be because nestling colonies of eiders are smaller in number and size and limited to the northern shore of the gulf, an area with a low density of potential observers.

The presence of HPAI H5N1 clade 2.3.4.4b viruses with 2 different genome constellations and the temporo-geographic distribution of the seals indicates that this outbreak was associated with >1 source of infection, an assertion supported by phylogenetic analyses. Some seal-derived sequences form monophyletic clades that exclude those from wild birds (e.g., samples FAV-0836-1, FAV-0836-2, and FAV-0836-6; Figure 5), suggesting a common source of infection for those seals. However, with the data currently available, we cannot determine if direct seal-to-seal transmission occurred. In addition, the absence of cases of HPAI H5N1 in seals from the St. Lawrence Estuary since the original outbreak (Canadian Wildlife Health Cooperative database, unpub. data) suggests that this condition has not become endemic in this population of marine mammals.

In conclusion, the infection of mammal species such as seals by HPAI H5N1 viruses raises concern about recent viral mutations making possible entry and replication within mammalian cells. From a human health perspective, such changes in viral host range warrant continued vigilance to detect a potentially deadly epidemic before its emergence. In addition, marine mammals, such as seals or other pinnipeds, might act as reservoirs for this virus, which could contribute to increasing risk for mutations and viral reassortment, favoring the infection of new mammal hosts. Therefore, monitoring the occurrence and molecular characteristics of this HPAI virus in populations of wild marine mammals is essential for assessing the public health risk associated with this emerging pathogen–host dynamic.

## References

[1] Fereidouni S, Munoz O, Von Dobschuetz S, De Nardi M. Influenza virus infection of marine mammals. EcoHealth. 2016;13:161–70. 10.1007/s10393-014-0968-1 25231137

[2] Anthony SJ, St Leger JA, Pugliares K, Ip HS, Chan JM, Carpenter ZW, et al. Emergence of fatal avian influenza in New England harbor seals. MBio. 2012;3:e00166–12. 10.1128/mBio.00166-12 22851656 PMC3419516

[3] Bodewes R, Bestebroer TM, van der Vries E, Verhagen JH, Herfst S, Koopmans MP, et al. Avian Influenza A(H10N7) virus-associated mass deaths among harbor seals. Emerg Infect Dis. 2015;21:720–2. 10.3201/eid2104.141675 25811303 PMC4378483

[4] Groth M, Lange J, Kanrai P, Pleschka S, Scholtissek C, Krumbholz A, et al. The genome of an influenza virus from a pilot whale: relation to influenza viruses of gulls and marine mammals. Infect Genet Evol. 2014;24:183–6. 10.1016/j.meegid.2014.03.026 24704761

[5] Reperant LA, Rimmelzwaan GF, Kuiken T. Avian influenza viruses in mammals. Rev Sci Tech. 2009;28:137–59. 10.20506/rst.28.1.1876 19618623

[6] van den Brand JMA, Wohlsein P, Herfst S, Bodewes R, Pfankuche VM, van de Bildt MWG, et al. Influenza A (H10N7) virus causes respiratory tract disease in harbor seals and ferrets. PLoS One. 2016;11:e0159625. 10.1371/journal.pone.0159625 27448168 PMC4957826

[7] Duignan PJ, Van Bressem MF, Cortés-Hinojosa G, Kennedy-Stoskopf S. Viruses. In: Gulland FMD, Dierauf LA, Whitman KL, editors. CRC handbook of marine mammal medicine. 3rd edition. Boca Raton (FL): CRC Press; 2018. p. 331–66.

[8] Shin DL, Siebert U, Haas L, Valentin-Weigand P, Herrler G, Wu NH. Primary harbour seal (*Phoca vitulina*) airway epithelial cells show high susceptibility to infection by a seal-derived influenza A virus (H5N8). Transbound Emerg Dis. 2022;69:e2378–88. 10.1111/tbed.14580 35504691

[9] Measures LN, Fouchier RAM. Antibodies against influenza virus types A and B in Canadian seals. J Wildl Dis. 2021;57:808–19. 10.7589/JWD-D-20-00175 34410421

[10] Berhane Y, Joseph T, Lung O, Embury-Hyatt C, Xu W, Cottrell P, et al. Isolation and characterization of novel reassortant influenza A(H10N7) virus in a harbor seal, British Columbia, Canada. Emerg Infect Dis. 2022;28:1480–4. 10.3201/eid2807.212302 35731188 PMC9239883

[11] Caliendo V, Lewis NS, Pohlmann A, Baillie SR, Banyard AC, Beer M, et al. Transatlantic spread of highly pathogenic avian influenza H5N1 by wild birds from Europe to North America in 2021. Sci Rep. 2022;12:11729. 10.1038/s41598-022-13447-z 35821511 PMC9276711

[12] Alkie TN, Cox S, Embury-Hyatt C, Stevens B, Pople N, Pybus MJ, et al. Characterization of neurotropic HPAI H5N1 viruses with novel genome constellations and mammalian adaptive mutations in free-living mesocarnivores in Canada. Emerg Microbes Infect. 2023;12:2186608. 10.1080/22221751.2023.2186608 36880345 PMC10026807

[13] Bevins SN, Shriner SA, Cumbee JC Jr, Dilione KE, Douglass KE, Ellis JW, et al. Intercontinental movement of highly pathogenic avian influenza A(H5N1) clade 2.3.4.4 virus to the United States, 2021. Emerg Infect Dis. 2022;28:1006–11. 10.3201/eid2805.220318 35302933 PMC9045435

[14] Canadian Food Inspection Agency. Highly pathogenic avian influenza in wildlife [cited 2023 May 16]. https://cfia-ncr.maps.arcgis.com/apps/dashboards/89c779e98cdf492c899df23e1c38fdbc

[15] Elsmo EJ, Wünschmann A, Beckmen KB, Broughton-Neiswanger LE, Buckles EL, Ellis J, et al. Highly pathogenic avian influenza A(H5N1) virus clade 2.3.4.4b infections in wild terrestrial mammals, United States, 2022. Emerg Infect Dis. 2023;29:2451–60. 10.3201/eid2912.230464 37987580 PMC10683806

[16] Vreman S, Kik M, Germeraad E, Heutink R, Harders F, Spierenburg M, et al. Zoonotic mutation of highly pathogenic avian influenza H5N1 virus identified in the brain of multiple wild carnivore species. Pathogens. 2023;12:168. 10.3390/pathogens12020168 36839440 PMC9961074

[17] Puryear W, Sawatzki K, Hill N, Foss A, Stone JJ, Doughty L, et al. Highly pathogenic avian influenza A(H5N1) virus outbreak in New England seals, United States. Emerg Infect Dis. 2023;29:786–91. 10.3201/eid2904.221538 36958010 PMC10045683

[18] Postel A, King J, Kaiser FK, Kennedy J, Lombardo MS, Reineking W, et al. Infections with highly pathogenic avian influenza A virus (HPAIV) H5N8 in harbor seals at the German North Sea coast, 2021. Emerg Microbes Infect. 2022;11:725–9. 10.1080/22221751.2022.2043726 35172704 PMC8890524

[19] Shin DL, Siebert U, Lakemeyer J, Grilo M, Pawliczka I, Wu NH, et al. Highly pathogenic avian influenza A(H5N8) virus in gray seals, Baltic Sea. Emerg Infect Dis. 2019;25:2295–8. 10.3201/eid2512.181472 31742519 PMC6874272

[20] Gamarra-Toledo V, Plaza PI, Gutiérrez R, Inga-Diaz G, Saravia-Guevara P, Pereyra-Meza O, et al. Mass mortality of sea lions caused by highly pathogenic avian influenza A(H5N1) virus. Emerg Infect Dis. 2023;29:2553–6. 10.3201/eid2912.230192 37916983 PMC10683807

[21] Ulloa M, Fernández A, Ariyama N, Colom-Rivero A, Rivera C, Nuñez P, et al. Mass mortality event in South American sea lions (*Otaria flavescens*) correlated to highly pathogenic avian influenza (HPAI) H5N1 outbreak in Chile. Vet Q. 2023;43:1–10. 10.1080/01652176.2023.2265173 37768676 PMC10588531

[22] Leguia M, Garcia-Glaessner A, Muñoz-Saavedra B, Juarez D, Barrera P, Calvo-Mac C, et al. Highly pathogenic avian influenza A (H5N1) in marine mammals and seabirds in Peru. Nat Commun. 2023;14:5489. 10.1038/s41467-023-41182-0 37679333 PMC10484921

[23] Geraci JR, Lounsbury V. Marine mammals ashore. A field guide for strandings. Galveston (TX, USA): Texas A&M Sea Grant Publications; 1993.

[24] Wilson DE, Mittermeier RA. Handbook of the mammals of the world, vol. 4: sea mammals. Barcelona (Spain): Lynx Edicions; 2014.

[25] Weingartl HM, Berhane Y, Hisanaga T, Neufeld J, Kehler H, Emburry-Hyatt C, et al. Genetic and pathobiologic characterization of pandemic H1N1 2009 influenza viruses from a naturally infected swine herd. J Virol. 2010;84:2245–56. 10.1128/JVI.02118-09 20015998 PMC2820904

[26] Spackman E, Senne DA, Myers TJ, Bulaga LL, Garber LP, Perdue ML, et al. Development of a real-time reverse transcriptase PCR assay for type A influenza virus and the avian H5 and H7 hemagglutinin subtypes. J Clin Microbiol. 2002;40:3256–60. 10.1128/JCM.40.9.3256-3260.2002 12202562 PMC130722

[27] Chrzastek K, Lee DH, Smith D, Sharma P, Suarez DL, Pantin-Jackwood M, et al. Use of Sequence-Independent, Single-Primer-Amplification (SISPA) for rapid detection, identification, and characterization of avian RNA viruses. Virology. 2017;509:159–66. 10.1016/j.virol.2017.06.019 28646651 PMC7111618

[28] Suchard MA, Lemey P, Baele G, Ayres DL, Drummond AJ, Rambaut A. Bayesian phylogenetic and phylodynamic data integration using BEAST 1.10. Virus Evol. 2018;4:vey016. 10.1093/ve/vey016 29942656 PMC6007674

[29] Kalyaanamoorthy S, Minh BQ, Wong TKF, von Haeseler A, Jermiin LS. ModelFinder: fast model selection for accurate phylogenetic estimates. Nat Methods. 2017;14:587–9. 10.1038/nmeth.4285 28481363 PMC5453245

[30] Minin VN, Bloomquist EW, Suchard MA. Smooth skyride through a rough skyline: Bayesian coalescent-based inference of population dynamics. Mol Biol Evol. 2008;25:1459–71. 10.1093/molbev/msn090 18408232 PMC3302198

[31] Rambaut A, Drummond AJ, Xie D, Baele G, Suchard MA. Posterior summarization in Bayesian phylogenetics using tracer 1.7. Syst Biol. 2018;67:901–4. 10.1093/sysbio/syy032 29718447 PMC6101584

[32] Caliendo V, Leijten L, van de Bildt M, Germeraad E, Fouchier RAM, Beerens N, et al. Tropism of highly pathogenic avian influenza H5 viruses from the 2020/2021 epizootic in wild ducks and geese. Viruses. 2022;14:280. 10.3390/v14020280 35215873 PMC8880460

[33] Klopfleisch R, Wolf PU, Uhl W, Gerst S, Harder T, Starick E, et al. Distribution of lesions and antigen of highly pathogenic avian influenza virus A/Swan/Germany/R65/06 (H5N1) in domestic cats after presumptive infection by wild birds. Vet Pathol. 2007;44:261–8. 10.1354/vp.44-3-261 17491066

[34] Keawcharoen J, Oraveerakul K, Kuiken T, Fouchier RA, Amonsin A, Payungporn S, et al. Avian influenza H5N1 in tigers and leopards. Emerg Infect Dis. 2004;10:2189–91. 10.3201/eid1012.040759 15663858 PMC3323383

[35] Reperant LA, van Amerongen G, van de Bildt MW, Rimmelzwaan GF, Dobson AP, Osterhaus AD, et al. Highly pathogenic avian influenza virus (H5N1) infection in red foxes fed infected bird carcasses. Emerg Infect Dis. 2008;14:1835–41. 10.3201/eid1412.080470 19046504 PMC2634621

[36] Mosnier A, Dispas A, Hammill MO. Spatial distribution and count of harbour seals (*Phoca vitulina*) and grey seals (*Halichoerus grypus*) in the Estuary and Gulf of St. Lawrence from an aerial survey conducted in June 2019. Can Tech Rep Fish Aquat Sci. 2023;3541:v–60. 10.3201/eid1412.080470

